# Prescribing practices for proton pump inhibitors among primary care physicians in England: an evaluation

**DOI:** 10.3399/BJGPO.2024.0059

**Published:** 2025-04-09

**Authors:** Kate Plehhova, Joshua Wray, Patricia Aluko, Scott Sutton, Jim McArdle, Anne Dawson, Cathal Coyle, Richard M Stevens

**Affiliations:** 1 Gastrointestinals, Reckitt Benckiser Healthcare Limited, Hull, United Kingdom; 2 Reckitt Benckiser Healthcare Limited, Hull, United Kingdom; 3 Interface Clinical Services, Leeds, United Kingdom; 4 Primary Care Society for Gastroenterology, Oxford, United Kingdom

**Keywords:** primary health care, proton pump inhibitors, deprescriptions

## Abstract

**Background:**

Proton pump inhibitors (PPIs), the most frequently prescribed drug class globally, are often overused.

**Aim:**

To assess PPI prescribing practice in England.

**Design & setting:**

Electronic medical record (EMR) evaluation from 62 primary care GP practices in England.

**Method:**

Adult patients on continuous PPI treatment (repeat prescription or ≥4 acute prescriptions 6 months before data extraction) were included (August 2021−June 2022) to compare PPI prescribing practices versus National Institute for Health and Care Excellence (gastro-oesophageal reflux disease [GORD] and dyspepsia management) and Medicines and Healthcare products Regulatory Agency (clopidogrel and PPI interaction) guidelines.

**Results:**

We identified 77 356 patients on continuous PPI treatment. The most common (68%) diagnosis recorded in patients’ EMRs and indicated for PPI use was gastroprotection, although 62% had no recorded indication. Of these 62% of patients, 40% had no medication review in the preceding year. Among those with diagnoses indicated for ≤3 months of PPI therapy (34%), 99% received their first PPI prescription ≥3 months previously. Of patients with diagnoses indicated for long-term treatment (4%), 41% had no medication review in the preceding year. Furthermore, 18% of patients using omeprazole or esomeprazole were also prescribed clopidogrel, and 19% of those prescribed treatments associated with gastrointestinal risk (*n* = 14 826) were not prescribed PPIs.

**Conclusion:**

This study shows that PPI prescribing in England is not in alignment with existing clinical guidelines and highlights the need for appropriate measures to increase awareness of overuse and support deprescribing where appropriate.

## How this fits in

In alignment with previous reports from the UK and other European countries, our findings reveal a sustained pattern of inappropriate (that is, not in line with clinical guidelines) PPI prescribing practices in England without systematic medication review. Considering these findings and the growing concerns over potential adverse effects associated with long-term PPI therapy, interventions aimed at promoting a more rational and controlled prescribing of PPIs will result in improved patient care.

## Introduction

Proton pump inhibitors (PPIs), such as omeprazole and lansoprazole, are a class of medications that inhibit stomach acid production.^1^ Since their introduction into clinical practice more than 30 years ago, PPIs have transformed the therapeutic landscape of acid-related disorders, including gastro-oesophageal reflux disease (GORD), Zollinger–Ellison syndrome, and peptic ulcers.^2,3^ They are among the most-prescribed class of pharmaceuticals worldwide.^4–7^ In England, omeprazole prescriptions tripled between 2006 and 2016, and more than 35 million prescriptions were issued in 2022–2023, making it the second most dispensed drug in the country.^8,9^ Although PPI prescribing prevalence has increased steadily over the years, the number of new starters has been relatively stable, suggesting that the observed increase in use stems from an increasing proportion of long-term treatments.^10^

PPIs are most effective in erosive conditions, such as erosive esophagitis, which represents about 30% of GORD cases.^11,12^ Most people with GORD (70%) have endoscopy-negative reflux disease, such as non-erosive reflux disease, reflux hypersensitivity, and functional heartburn, where PPIs provide less symptom relief, and short-term treatment (4 or 8 weeks) is recommended.^11–13^ However, PPI use often extends beyond the recommended indication and treatment duration, likely because they are cost-effective, easily available, and considered relatively safe.^2,14^ Continued use of non-steroidal anti-inflammatory drugs (NSAIDs) and aspirin, especially in the older population, also promotes PPI co-prescriptions for gastroprotection.^5^ Further, the reduced frequency of systematic follow-up by doctors in overburdened healthcare systems allows patients to use repeat prescriptions indefinitely.^2^ In the UK, almost 40% of patients prescribed PPIs continue treatment for over a year and 10%, for over 5 years.^15^ It has also been reported that about 20% of patients on PPIs or other gastroprotectants, such as histamine-2 receptor antagonist (H2RA), have no recorded indication for their use.^15^

PPI overuse is clinically concerning given the evidence regarding potential risks associated with their long-term use, including bone fractures, kidney disease, enteric infections (especially *Clostridioides difficile*), and community-acquired pneumonia.^16^ Therefore, clinical practice guidelines in some countries have recommended deprescribing PPIs.^13,14,17,18^ The National Institute for Health and Care Excellence (NICE) recommends a 4-week treatment period for dyspepsia, 4 or 8 weeks for GORD, and annual medical reviews for patients requiring long-term therapy to step down or stop treatment.^13^ In addition, the Medicines and Healthcare products Regulatory Agency (MHRA) discourages the concurrent use of clopidogrel with omeprazole and esomeprazole.^19^

The present study reviewed the electronic medical records (EMRs) of patients registered at 62 primary care GP practices in England to assess doctors’ prescribing practices for PPI in the context of reflux management. It represents the first phase of a wider quality-improvement project aimed at supporting appropriate use of PPIs among primary care practitioners in England.

## Method

### Study design and data sources

This was a retrospective review of anonymised EMRs extracted from the EMIS Web and TPP SystmOne platforms, provided by Interface Clinical Services (UK), an IQVIA company (henceforth referred to as Interface).

EMIS Web and TPP SystmOne are the two main EMR systems used by GPs in the UK, cumulatively covering approximately 90% of UK practices.^20^ They include detailed information on patient demographics, medical diagnosis, biochemistry data, GP prescriptions, and secondary care data, whereby GPs manually transfer secondary care information onto the EMRs.

Primary care GP practices were selected across England based on the following two key criteria: those covered by the Interface network; and having an existing dyspepsia management pathway. Interface pharmacists briefed GP practices regarding the purpose of the study and informed practices that agreed to participate were included on a first-come, first-served basis. Network-level delivery was prioritised. Based on this approach, consent for data extraction was obtained from 62 primary care GP practices, covering 38 primary care networks within 19 clinical commissioning groups across England. Data were extracted between 3 August 2021 and 7 June 2022, by running predefined queries designed to collect information on patient demographics, medical diagnoses, and GP prescriptions, including issue dates, prescribed drugs, daily doses, quantities, and pack sizes.

### Study populations

**Figure 1.**
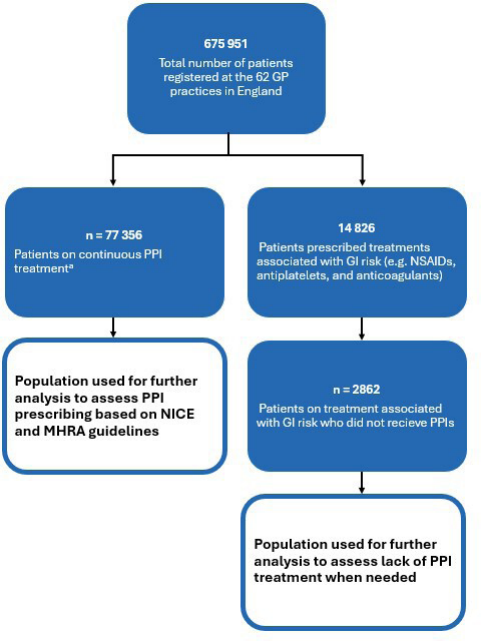
Population selection process. ^a^Patients with a repeat prescription or receiving ≥4 acute prescriptions in the 6 months before data extraction. GI = gastrointestinal. MHRA = Medicines and Healthcare products Regulatory Agency. NICE = National Institute for Health and Care Excellence. NSAIDs = non-steroidal anti-inflammatory drugs. PPI = proton pump inhibitor

In total, 62 NHS primary care GP practices consented to participate, providing data for 675 951 adults (≥18 years). From this group, we identified patients on continuous PPI treatment (that is, those who received a repeat prescription at any time before data extraction or who received ≥4 acute prescriptions in the preceding 6 months) (Figure 1). This population was further analysed to assess PPI prescribing practices for reflux management. To assess the proportion of patients not prescribed PPIs despite needing them, we identified at-risk patients who received prescriptions for treatments associated with gastrointestinal (GI) risk (NSAIDs, antiplatelets, and anticoagulants) and were not on gastroprotectants (Figure 1). PPIs identified from the *British National Formulary* included the following: esomeprazole; lansoprazole; omeprazole; pantoprazole; and rabeprazole.

### Study outcomes to assess PPI prescribing practices for reflux management

**Figure 2.**
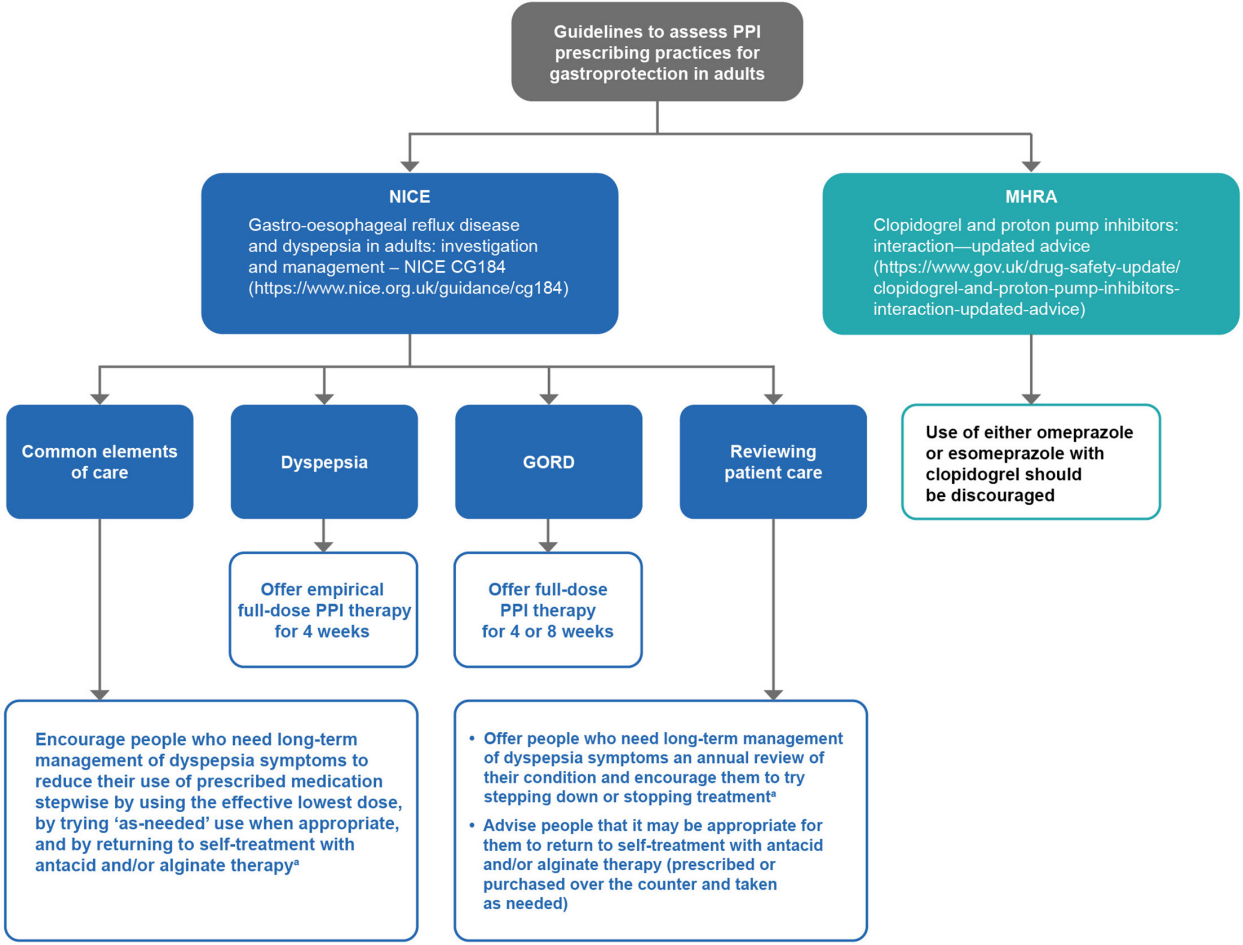
Guidelines to assess PPI prescribing practices for gastroprotection in adults. Adapted from NICE clinical guideline CG184 (2019) sections 1.2, 1.4, 1.5, and 1.6^13^ and the MHRA’s updated advice on the interaction between clopidogrel and proton pump inhibitors (December 2014)^19^. ^a^Unless there is an underlying condition or comedication that needs continuing treatment. GORD = gastro-oesophageal reflux disease. MHRA = Medicines and Healthcare products Regulatory Agency. NICE = National Institute for Health and Care Excellence. PPI = proton pump inhibitor

PPI prescribing was assessed for patients on continuous PPI treatment (Figure 1) based on the NICE clinical guideline (CG184) on management of GORD and dyspepsia in adults and the MHRA drug-safety advice on clopidogrel use with PPIs (Figure 2).^13,19^

Using patients’ clinical records and NICE guidelines, we initially evaluated potential reasons for PPI use in England by identifying recorded diagnoses of diseases for which PPIs are indicated: medication-induced dyspepsia, GORD, GI cancers, Zollinger–Ellison syndrome, Barrett’s oesophagus, oesophageal strictures, hiatus hernia, oesophagitis, GI ulceration, and dyspepsia.^13^ We also examined the presence of indications associated with prescriptions to evaluate the proportion of patients who had a record of the actual reasons for being prescribed PPIs. Finally, we described PPI prescribing practices deviating from NICE guidelines for GORD and dyspepsia management or the MHRA drug-safety advice on concurrent clopidogrel and PPI use (Table 1).

**Table 1.** Study outcomes evaluated among patients on continuous proton pump inhibitor treatment (that is, patients receiving repeat prescription or ≥4 acute prescriptions within the 6 months before data extraction)

PPI prescribing practices deviating from recommendations reported in English clinical guidelines	Outcome	Definition
Concurrent PPI and clopidogrel treatment	Among patients on concurrent PPI and clopidogrel treatment, patients who are on omeprazole or esomeprazole	As mentioned in the MHRA clopidogrel and PPI interaction-drug safety advice^19^
Continuous PPI treatment on a higher dose than necessary	Among patients on continuous PPI treatment, proportion (%) on a full dose, low dose, and double dose of PPI	As defined in NICE CG184, Appendix A^13^
Continuous PPI treatment without a recorded diagnosis of a disease for which long-term use (>3 months) of PPIs is indicated^13^	Among patients on continuous PPI treatment, proportion (%) of those diagnosed with a disease for which long-term PPI treatment is indicated based on NICE guidelines	Recorded SNOMED codes for oesophagitis, Barrett’s oesophagus, chronic NSAID use and bleeding risk, oesophageal strictures, and Zollinger–Ellison syndrome at any time before data extraction
Continuous PPI treatment for conditions for which short-term treatment (≤3 months) with PPIs is indicated	Among patients on continuous PPI treatment, proportion (%) of those diagnosed with a disease for which short-term PPI treatment is indicated based on NICE guidelines and: time (months) since first prescription number of prescriptions received in the previous 12 months	Recorded SNOMED codes for dyspepsia, GORD, and GI ulcer Time in months since the issue of the first PPI prescription in the patient’s entire medical history Number of prescriptions in the 12 months before data extraction
Lack of medical review to change, reduce, or stop PPI treatment if needed	Overall and for all groups described above, proportion of patients who did not have: an annual medical review further GI assessment	No record of annual medical review at any point after first prescription of PPIs No record of investigations and/or referrals for GI assessments at any point after first prescription of PPIs

a Please refer to Supplementary Table S1 for codes. GI = gastrointestinal. GORD = gastro-oesophageal reflux disease. NSAID = non-steroidal anti-inflammatory drug. MHRA = Medicines and Healthcare products Regulatory Agency. NICE = National Institute for Health and Care Excellence. PPI = proton pump inhibitor. SNOMED= Systematised Nomenclature of Medicine.

### Data analysis

Anonymised data from each practice were pooled into one dataset. Microsoft Office 365 was used to process the data and generate output tables and figures. Descriptive statistics (*n*, %) were used to summarise the results.

## Results

### Study population

Among 675 951 registered patients, 77 356 (11%) met the criteria for continuous PPI treatment (Figure 1). Further, 14 826 patients were prescribed treatments associated with GI risk, among which 2862 patients (19%) were not prescribed gastroprotective treatment, including PPIs. Table 2 shows the characteristics of patients on continuous PPI treatment and those not on PPI treatment when needed.

**Table 2.** Demographics and characteristics of study populations

Characteristic		Patients on continuous PPI treatment (*n* = 77 356)	Patients not on PPI when prescribed treatment associated with GI risk (*n* = 2862)
		*n* (%)	*n* (%)
Sex	Male	35 141 (45%)	1230 (43%)
	Female	42 215 (55%)	1632 (57%)
Age group (years)	18−29	1695 (2%)	371 (13%)
	30−39	3977 (5%)	474 (17%)
	40−49	7172 (9%)	558 (19%)
	50−59	14 602 (19%)	702 (25%)
	60−69	18 175 (23%)	438 (15%)
	70−79	19 231 (25%)	215 (8%)
	80−89	10 261 (13%)	85 (3%)
	≥90	2224 (3%)	6 (0.2%)
Frailty status	Non-specific	304 (0.4%)	3 (0.1%)
	Mild	6394 (8%)	75 (3%)
	Moderate	6254 (8%)	48 (2%)
	Severe	3378 (4%)	20 (0.7%)
Number of currently prescribed items (polypharmacy)	1−3	17 288 (22%)	1177 (41%)
	4−6	20 887 (27%)	658 (23%)
	7−9	17 102 (22%)	338 (12%)
	≥10	21 148 (27%)	263 (9%)
Among patients on ≥10 medications, those who had had no medical review in the preceding 12 months		6722 (32%)	95 (36%)

ab Maximum age 104 years and 99 years, respectively. Values may not add up to 100% owing to some patients not fulfilling the characteristic threshold criteria. GI = gastrointestinal. PPI = proton pump inhibitor

### PPI prescribing practices among patients on continuous PPI treatment

Based on patients’ EMRs and NICE guidelines, the predominant indication for PPI therapy was gastroprotection from dyspepsia-causing medication, found in 68% of patients on continuous PPI treatment (52 253/77 356), followed by GORD in 36% of patients (27 995/77 356), and dyspepsia in 27% of patients (20 938/77 356) (Table 3). Most patients (52 211/77 356, 67%) were prescribed full-dose PPI therapy; 18% of patients (13 763/77 356) were given low-dose therapy, and 15% of patients (11 382/77 356), double dose. Further, 5449 patients were co-prescribed clopidogrel, of which 18% of patients (*n* = 989) were using omeprazole or esomeprazole.

**Table 3.** Potential indications for proton pump inhibitors in patients’ electronic medical records

	*n* = 77 356
**Indication^a^**	***n* (%)**
Dyspepsia-causing medication	52 253 (68%)
GORD	27 995 (36%)
Dyspepsia	20 938 (27%)
Hiatus hernia	15 218 (20%)
Oesophagitis	12 508 (16%)
GI ulceration	4115 (5%)
Barrett’s oesophagus	2753 (4%)
Oesophageal strictures	353 (0.5%)
GI cancers	283 (0.4%)
Zollinger–Ellison syndrome	1 (0.0%)

a For some patients, more than one indication was recorded. GI = gastrointestinal. GORD = gastro-oesophageal reflux disease

**Figure 3.**
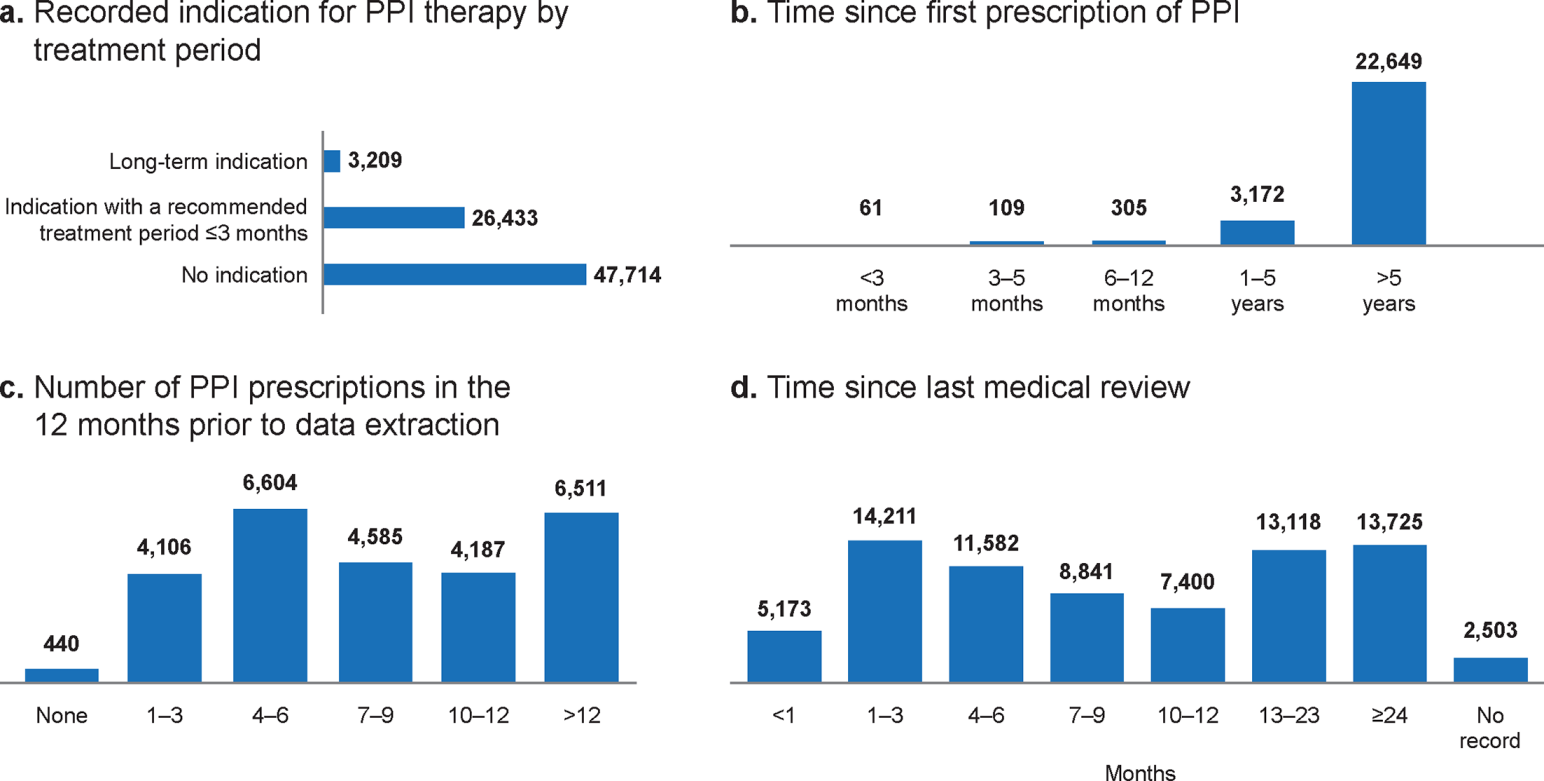
Assessment of proton pump inhibitor (PPI) use among patients on continuous PPI treatment

Most patients (47 714/77 356; 62%) had no indication for PPI therapy associated with their prescriptions (Figure 3a), and 40% of them (19 105/47 714) had not received a medication review within the previous 12 months. Overall, 34% of patients (26 433/77 356) on continuous PPI treatment had recorded indications for short-term (≤3 months) PPI therapy, as recommended by NICE guidelines for dyspepsia, GORD, and any GI ulceration (Figure 3a). However, 99% of these patients received their first PPI prescription ≥3 months before, and 86% received it >5 years before (Figure 3b). Further, 40% of them (10 698/26 433) had≥10 PPI prescriptions in the previous year (Figure 3c).

Only 4% of patients on continuous PPI treatment (3209/77 356) had a recorded indication for long-term therapy (Figure 3a), namely, Barrett’s oesophagus, chronic NSAID use and bleeding risk, severe oesophagitis, oesophageal strictures, and Zollinger–Ellison syndrome. Forty-one per cent of this subset (1316/3209) had no record of medication review in the preceding year.

Overall, more than one-third of the patients on continuous PPI treatment (29 346/77 356; 38%) had no record of a medication review in the year preceding data extraction (Figure 3d), and almost half of them (37 508/77 356; 48%) had no record of further GI assessments in the preceding 5 years (Supplementary Table S2).

## Discussion

### Summary

This study shows that patients on continuous PPI therapy often have no recorded indications, are rarely diagnosed with diseases that justify long-term PPI use, and lack follow-up or medication reviews. These practices lead to deviations from existing guidelines on PPI use, which are likely driven by the need for symptom relief.

### Strengths and limitations

This study provides clinically relevant insights into real-world PPI prescribing practices in England by analysing EMRs of a large sample of 675 951 registered patients at 62 practices countrywide. The population-selection criteria were the same for all practices, and patients from different practices presented similar demographics. Thus, we could confidently identify specific trends in PPI prescribing at the included primary care GP practices.

However, some limitations should be considered when interpreting the results. First, the sample of practices represented just below 1% of all primary care GP practices in England at the time of the study.^21^ Since Interface only works in~2000 of the~6000 GP practices in the UK, it only has access to around one-third of all GP practices, and the service is only available to practices that use EMIS Web or TPP SystmOne as clinical systems. Further, the study allowed for approximately 6 months of practice recruitment, so not all practices could be contacted to offer the service within this timeframe, and network practices with readily available access were more likely to be recruited. This poses some bias, in that the recruited practices would likely have had prior exposure or experience of working with Interface on other therapy review programmes aimed at improving patient care. Additionally, the sample of patients was not evenly distributed across the different practices, with some clinics contributing more patients than others. Thus, our results may not necessarily apply to all GP clinics in the country.

Second, this study is a descriptive analysis of the data, and no statistical comparisons were conducted. No information on ethnicity was collected. Furthermore, as for all EMR analyses, this study had limitations inherent to data collection, such as missing and/or incomplete data and inability to capture PPI prescriptions in hospital settings and those purchased over the counter. The datasets provided limited information on adverse events, hospitalisations, refractory symptoms, and investigations (for example, which patients had a positive diagnosis for GORD), and therefore, this information is not reported in this study. Data on adverse events could have provided additional insights into the risks associated with PPI over-prescribing and should be evaluated in future analyses. Notably, information on patients’ symptoms and response to treatment logged within the unstructured medical notes could not be captured in this study. It is also important to acknowledge that coded medication reviews may not specifically capture PPI reviews, as some clinical practices may only consider certain medications and not others when conducting a review. Therefore, the frequency of PPI reviews may have been underestimated in this study, especially if patients were also on other medications. Finally, as the current study was conducted during the COVID-19 pandemic, the plausible impact on overall prescribing and standard of care practices cannot be undermined.

### Comparison with existing literature

Of all registered patients included in our study, 11% were on continuous PPI therapy, which aligns with reports of the increase in PPI use from 0.2% to 14.2% in the UK between 1990 and 2018.^15,22^

The predominant potential indication for PPIs was gastroprotection from dyspepsia-causing medication, followed by GORD and dyspepsia. These findings align with a global systematic review spanning three decades, where the most prevalent indication was prophylaxis, followed by GORD and dyspepsia.^10^

Despite national guidelines recommending full-dose therapy for short durations (4 or 8 weeks) and a step down thereafter, 15% of the patients in our study were on a double dose of PPI. This trend was also noted in the global systematic review, where most PPI users (63.7%) were prescribed higher doses than the indicated daily dose.^10^ Further, almost 18% of the patients on PPI–clopidogrel comedication were on omeprazole or esomeprazole, despite this being contraindicated in the MHRA guidelines.^19^

Around 62% of patients on continuous PPI treatment in our study had no recorded indication associated with their prescriptions. This observation is important, as it suggests that PPIs are inappropriately used, although it is important to note that this phenomenon has also been reported for other drugs in the UK.^23^ This proportion is higher compared with that in the cross-sectional study conducted in 1990−2018, where 20% of patients on PPI and H2RA had no recorded indication for use.^15^ Similarly, the above-mentioned global review found that approximately 15% of PPI users had an unclear or no recorded indication for use.^10^

The present study found that 34% of patients on continuous PPI therapy had a diagnosis for a disease indicated for ≤3 months of PPI treatment, which is in accordance with NICE guidelines. Yet, almost all of them (99%) had received their first prescription ≥3 months before data extraction, and 40% of them had received ≥10 PPI prescriptions in the prior 12 months. In addition, despite national guidelines recommending annual reviews for patients on long-term PPI therapy, 41% of patients diagnosed with diseases recommended for long-term therapy by NICE had no medication review in the preceding year.^13^ This trend was also noted in the UK-based study evaluating prescribing patterns between 1990 and 2014, where the authors found that 26.7% and 3.9% of the study population remained on PPI therapy for≥1 and 5 years, respectively.

### Implications for practice

While PPIs are effective for managing acid-related disorders, their excessive and inappropriate usage has become a global phenomenon.^10^ The UK has witnessed a continuous rise in the number of PPI items dispensed, with more than 73 million dispensed during 2022–2023 in England alone, at a total cost of over 192 million GBP.^24^

The NICE guidelines for GORD and dyspepsia management in adults were updated in 2019 and recommend annual patient reviews to re-assess long-term PPI therapy and to encourage patients to step down or off treatment. To this end, NICE recommends using the lowest dose of PPI on an ‘as-needed’ basis and using antacids and/or alginates if required.^13^ However, despite recently published evidence on PPI use, NICE has not yet published specific guidelines on PPI deprescribing.

According to NHS records, omeprazole was the third most dispensed chemical item in 2019 (31.9 million), and it has moved up to the second position in 2022–2023, with over 35 million items dispensed.^8,25^ From a study indicating a plateau in PPI prescribing intensity in the UK over the past 15 years, but an increase in the prevalence of PPI use, it can be deduced that a high proportion of patients continue long-term PPI therapy.^15^ The COVID-19 pandemic has exacerbated the pressure on an already-stressed NHS, and the strain on primary care GP practices, which were at the forefront of pandemic management, is particularly high.^26^ Improvements in prescribing practices and better alignment with clinical guidelines may be expected in NHS primary care once these issues are resolved. In addition, clinical pharmacists have been recruited into general practice under the NHS since 2020; patients may benefit from this through regular structured medication reviews, especially those on multiple concurrent medications.^27^ Patient experiences highlighted concerns regarding long-term PPI therapy in a recent study, but this was not reflected in GPs’ prescribing patterns.^28^ Thus, services to assist patients with systematic medication review and deprescribing are required in primary care settings.

The present study also identified 2862 patients who were prescribed treatments associated with GI risk but not gastroprotection. A review of this patient population could help assess the appropriate gastroprotection needed for such at-risk patients.

In conclusion, the present study confirmed that PPI prescribing in England often deviates from national guidelines. Collectively, the findings highlight the urgent need for actions to increase awareness and educate healthcare professionals on appropriate use of PPIs to support deprescribing or treatment cessation as needed.

## Data

The dataset relied on in this article is available from the corresponding author on reasonable request.
